# Period of the day drives distinctions in the taxonomic and functional structures of reef fish assemblages

**DOI:** 10.1111/jfb.70228

**Published:** 2025-09-13

**Authors:** Marcos B. Lucena, Thiago C. Mendes, Cesar A. M. M. Cordeiro, Carlos E. L. Ferreira

**Affiliations:** ^1^ Reef Fish Ecology and Conservation Lab, Departamento de Biologia Marinha Universidade Federal Fluminense Niterói Brazil; ^2^ Laboratório de Ciências Ambientais Centro de Biociências e Biotecnologia Universidade Estadual do Norte Fluminense Campos dos Goytacazes Rio de Janeiro Brazil

**Keywords:** community structure, diurnal, nocturnal, reef fish, subtropical reef, survey, visual census

## Abstract

Circadian processes are key drivers of animal behaviour, influencing patterns of activity, resource partitioning and competition avoidance. Studies evaluating circadian changes on the structure of marine assemblages are lacking, especially for reef fish. Evaluating the changes imposed by the day–night cycle on the structure and functioning of these assemblages is of critical importance to understand the differences between the diurnal and nocturnal components, as well as the resources they exploit, to better model and manage them. Here, we assessed the fish fauna using an underwater visual census conducted at the same sites during both day and night on a subtropical rocky reef in the Southwestern Atlantic (SWA), aiming to evaluate differences in the community and functional structure of these assemblages. A total of 242 transects were conducted across three sites, with 104 during the day and 138 at night. Fish richness, density, biomass and functional richness were higher in the diurnal period, whereas nocturnal assemblages exhibited higher taxonomic distinctiveness and functional divergence. Both richness and density of nocturnal assemblage, as well as richness and taxonomic distinctness index of the diurnal assemblage, were influenced by depth. The species in each assemblage exhibited different combinations of traits. As expected, diurnal assemblages were richer and occupied a larger proportion of the multidimensional trait space. Body size was comparatively larger for the diurnal assemblage, evidencing niche partitioning based on ontogeny. These results indicate significant differences in the structure of diurnal and nocturnal reef fish assemblages, as expected, driven by resource use, space use and predator avoidance. Fish play a central role in many key processes within reef systems, in addition to their importance for human nutrition and cultural services. Therefore, understanding the components and diversity of nocturnal assemblages is essential to address the significant knowledge gap to understand their role in reef energetics and demand proper management.

## 1 INTRODUCTION

Earth rotation plays a major role in species ecology and behaviour by determining a series of rapid changes throughout the 24‐h cycle. The dynamics of the circadian process imposes changes, causing species to partition and exploit time as an ecological resource, influencing their behaviour and activity (Nagelkerken et al., 2000). The period of activity in which animals are more active is affected by several factors, both biotic, such as the influence of competitors or predators (Hammerschlag et al., 2010), and abiotic, such as the weather and climatic conditions (Gaston, 2019).

In the wild, reef fishes spend most of the day either foraging or avoiding predators (Helfman, 1986). Fish can be classified as diurnal, nocturnal or crepuscular (Helfman, 1978), as their adaptations optimize activity under specific light conditions, often reducing efficiency in different lighting environments (Schmitz & Wainwright, 2011). The period of the day in which fish are active has implications on the functional roles species play on ecosystems and is largely driven by either seeking for food and predator avoidance (Helfman, 1986). A small number of fish in tropical assemblages lack a particular period or peak in activity, and a large fraction of high‐latitude fishes are arrhythmic in their activity (Miiller, 1978). In general, the period of activity in reef fishes has phylogenetic roots, often at the family level (Helfman, 1986). Opportunistic behaviour, however, can make some fish feed during both day and night if food is available (Helfman, 1986). This temporal flexibility may mask the effects of predation risk, competition or ontogenetic shifts, while it may reflect evolutionary entrainment that allows the species to expand into different reef habitats (Fox & Bellwood, 2011).

It is well established that, at local scale, benthic cover (Ferreira et al., 2001; Longo et al., 2014), wave action (Ferreira et al., 2001), water temperature (Cordeiro et al., 2015; Guidetti & Boero, 2002), reef structural complexity (Brokovich et al., 2006; Dominici‐arosemena and Wolff, 2006) and depth (Cordeiro et al., 2015; Hernández‐Landa et al., 2015) are some of the major habitat driver influencing patterns of fish richness, density and biomass during daylight. Also at the local scale, factors such as species interactions – particularly those related to feeding – can influence the structure of fish assemblages (Almany, 2003). At night, these drivers are likewise expected to affect the distribution of nocturnally active fish assemblages.

Few studies have examined fish assemblage composition at night or comparatively between day and night (Azzurro et al., 2007; Brewin et al., 2016; Hinojosa et al., 2020; Jones et al., 2024). Among these, none has specifically analysed the influence of environmental drivers on nocturnal species during their active period, nor how these factors shape their functional ecology. There is a need to expand the studies on the conservation of reef environments beyond the knowledge of the distribution of species, incorporating knowledge of functional attributes (Mouillot et al., 2014; Schiettekatte et al., 2022).

For instance, the trophic impact of a species depends on its foraging activity, that is, which prey items it targets (diet), and when (period of activity) and where they are on reefs (Mouillot et al., 2014). Decreasing in the reef quality environment can lead to loss of biodiversity and associated ecological functions (Micheli & Halpern, 2005; Villeger et al., 2010). For example, overfishing of top predators can cause the entire trophic chain to break down and change energetic pathways (Mora et al., 2011).

The aim of this study was to compare the assemblage composition (identity and attributes) of diurnal and nocturnal reef fishes to evaluate changes between day and night. We also evaluate if any detected change would be reflected in functional arrangement and coupled attributes.

## 2 METHODS

### 2.1 Study site

Located in southeastern Brazil, the Arraial do Cabo region features an isthmus and four surrounding islands, characterized by extensive subtropical rocky shores and average water temperatures around 22°C (Cordeiro et al., 2020). Three sites with similar physiographic, benthic composition and fish assemblages (Cordeiro et al., 2015) were chosen to compare the taxonomic and functional structure of diurnal and nocturnal reef fish assemblages. All sites are part of the Marine Extractive Reserve of Arraial do Cabo, Rio de Janeiro state, Brazil (22°57′57″ S, 42°1′40″ W), which consists of a multiuse reserve where only local fishermen are allowed to exploit resources. No‐take areas are absent, and general enforcement is limited. Local reefs consist of shallow (up to 12 m) granitic rocky shores mainly covered with epilithic algae, zoanthids, sponges and few coral species (Cordeiro et al., 2020; Ferreira et al., 2001; Rogers et al., 2014). The region lacks significant freshwater inputs, with low precipitation (<850 mm year^−1^, INMET, 2020) resulting in good visibility (~ 8 m) almost all year around.

### 2.2 Survey technique

The fish assemblage was sampled using underwater visual census (UVC) using random 20 × 2 m strip transects at each study site (Figure S1). This method was chosen because it has been widely and effectively used to sample reef fish throughout the Brazilian province (Lucena et al., 2021; Morais et al., 2017). Both large (>10 cm) and small/cryptic (<10 cm) fishes were recorded using the same transect: larger, more mobile individuals were recorded during the initial deployment of the transect line, whereas small and cryptic species were recorded on the swim back. All individuals were identified to the lowest possible taxonomic level, and their sizes [total length (TL)] were estimated to the nearest centimetre (Morais et al., 2017). In our study, a species was considered ‘active’ during a given period (day or night) if individuals were observed exhibiting behaviours such as swimming in the water column, foraging, hovering over the substrate or displaying territorial or social interactions during the transect survey. Additionally, we referred to information available in the literature regarding species‐specific diel activity patterns to support classification when direct behavioural observations were limited. A total of 242 transects were conducted across three sites, with 104 during the day and 138 at night. Surveys were performed by a team of six trained divers over a 5‐month period. Although both day and night samplings were performed at the same locations, they were not always paired within a single 24‐h cycle due to logistical and environmental constraints.

### 2.3 Biomass estimates and taxonomic distinctness index

Biomass (*M*) was estimated for each individual fish using length–mass relationships, *M = a*. *L*_*T*_^*b*^, in which *L*_*T*_ is total length, and the parameters *a* and *b* are species‐specific constants derived from references in FishBase (Froese & Pauly, 2018). In cases where species coefficients were not available, coefficients of congeneric species, which were either phylogenetically or morphologically similar, were used. Priority was given to allometric parameters from local or regional studies to avoid large populational differences.

For each UVC, we calculated the taxonomic distinctness index (Clarke & Warwick, 1998), which is the average taxonomic ‘distance’ between any two organisms chosen at random from the sample. This distance can be visualized as the length of the path connecting these two species, traced through a Linnaean classification from references in FishBase (Froese & Pauly, 2018) of the full set of species involved (Clarke & Warwick, 1998).

### 2.4 Sampling design

To evaluate the reef fish assemblage composition, the survey design consisted of three factors: period (two levels, fixed: diurnal and nocturnal), site (random, three levels) and depth (continuous, ranging from 1.5 to 15 m). We avoided sampling in the twilight (1 h before and 1 h after the sunset) due to the intense activity of both diurnal and nocturnal species in this period, which could influence our results (Helfman, 1986; Rickel & Genin 2005). Diurnal sampling was conducted between 08:00 AM and 12:00 PM, and nocturnal sampling between 19:00 PM and 22:00 PM. These time windows refer to the overall sampling periods, with each individual UVC lasting approximately 6 min. During night sampling, each diver used a pair of flashlights (LED lamps, 1200 lumens, 6500 K): one hand‐held to illuminate the field of view and a second worn as a headlamp. The lights had no additional colour filters, and this configuration has been shown not to affect fish behaviour (Lucena et al., 2021). Sampling at night was restricted only to those species that were active during sampling. We excluded all detectable species that were inactive, such as parrotfishes. Species active in both periods (e.g., *Haemulon aurolineatum*) and crepuscular predators (e.g., *Mycteroperca acutirostris*) were considered in both diurnal and nocturnal samples.

To compare the functional structure of diurnal and nocturnal reef fish assemblages, we classified all observed species into six ecological traits (Quimbayo et al., 2021): (1) home range, (2) size group, (3) level in the water, (4) size class, (5) spawning type and (6) diet. Home range was coded using three ordered categories: sedentary or territorial (uses <100 m^2^), mobile (uses >100 m^2^ or travels among reef areas), very mobile (species that frequently change reefs or travel daily distances over the reef). Group size was coded using five ordered categories: solitary, pairing, small groups (2–20 individuals on average), medium groups (21–50 individuals on average), large groups (> 50 individuals on average). Level in the water was coded in three ordered categories: bottom, low (species that live slightly above the bottom), high (species that spend most of their activity high above the bottom). Size class was ordered in six ordered categories: 0–7, 7.1–15, 15.1–30, 30.1–50, 50.1–80 and >80 cm. Spawning was coded in four ordered categories: attach (attach the eggs to objects), demersal (deposit the eggs directly on the substrate), live (birth without external larval stage) and oral incubation (parental care when the fish keep the eggs in their mouth until they are fully developed). Diet was classified into seven categories based on the main items consumed by each species: herbivore‐detritivore (feeding mainly on the epilithic algal matrix), macroalgal feeders (feeding predominantly on macroalgae or seagrass), sessile invertebrates feeders (feeding predominantly on sessile benthic invertebrates), mobile benthic invertebrate feeders (feeding predominantly on mobile benthic invertebrates), planktivores (feeding predominantly on invertebrates in the water column, including zooplankton), omnivores (feeding on a range of organisms, animal or plant material) and piscivores (feeding predominantly on fishes but may also include cephalopods) (adapted from Quimbayo et al., 2021).

### 2.5 Statistical analyses

The effects of period of activity and depth on species richness, total density, biomass and taxonomic distinctness were investigated using mixed‐effects models with site as a random factor. Species richness and density were modelled using generalized linear mixed models (GLMM) with a Poisson distribution, biomass was modelled with a GLMM using a Tweedie distribution and taxonomic distinctness was modelled using a linear mixed model (LMM) with a Gaussian distribution. In all models, site was included as a random effect to account for potential spatial variability among sampling locations. For species richness, density and biomass, the random effect of site showed low‐to‐moderate variability, indicating some spatial structure in these metrics (Table S1). The taxonomic index applied was Δ^+^ (Dplus), following the recommendations of Clarke and Warwick (2001). To test whether the period of activity (fixed factor), depth (fixed factor) and site (random factor) influenced the diurnal and/or nocturnal composition of reef fish assemblages, a permutational multivariate analysis of variance (PERMANOVA) was performed using 999 permutations in Primer 7 (PERMANOVA add‐on). The analysis used square‐root transformed abundance data and Bray–Curtis similarity distance followed by a non‐metric multidimensional scaling (nMDS). An analysis of variance (ANOVA) was performed to compare fish sizes between the two periods. To compare the diurnal and nocturnal fish functional space, a dissimilarity matrix of species traits was computed using the Gower distance, which allows mixing different types of variables, while giving them equal weight (Legendre & Legendre, 1998). A principal co‐ordinates analysis (PCoA) was then performed using this distance matrix, and the first four principal axes were retained to build a multidimensional functional space (Mouillot et al., 2014).

The functional diversity was assessed using four functional indices: functional richness (functional space occupied by the community), functional evenness (regularity in the distribution of species abundances or biomass in the functional space), functional divergence (divergence in the distribution of biomass in the functional volume) and functional dispersion (how biomass is distributed within the volume of functional trait space occupied by species). Functional richness (FRic) for each community was measured as the volume inside the convex hull occupied by species of the community (Mouillot et al., 2013). For functional evenness, we computed functional evenness (FEve) with a minimum spanning tree (MST)‐based metric that varies from 0 to 1, with a value of 1 corresponding to a completely even distribution of abundance or biomass across trait space (Villéger et al., 2008). We weighted FEve using biomass (instead of number of individuals) to best reflect the functional response of the community (Villéger et al., 2008). Functional dispersion measure (FDis) quantifies the functional variation in reef fish assemblages by combining the relative biomass of species and functional traits (Laliberte & Legendre, 2010). FDis was calculated as the average distance of individual assemblages to the group weighted centroid in a multivariate functional trait space and is independent of species richness (Laliberte & Legendre, 2010). For functional divergence (FDiv), we followed Villéger et al. (2008) to calculate how species biomass diverges from the centre of the functional space. Differences between diurnal and nocturnal functional diversity indices were assessed using LMM with a Gaussian distribution. For most functional diversity indices (FRic and FDis), the random effect of site contributed only marginally to the overall variance. For FEve and FDiv, however, the models resulted in singular fits, indicating that the random effect of site had essentially zero variance and did not contribute to explaining the data. These models were retained for consistency across analyses but interpreted with caution regarding spatial effects (Table S1). Analyses were performed using R software (R Core Team, 2017), and the models were fitted using the ‘lme4’ package or PRIMER. GLMMs were conducted in R using the ‘lme4’ package. PERMANOVA analyses were carried out in PRIMER (with the PERMANOVA+ add‐on), using 999 permutations, square‐root transformed abundance data and Bray–Curtis similarity. (Oksanen et al., 2019); and functional indices were calculated using the ‘dbFD’ function from the ‘FD’ package (Laliberté et al., 2014).

This study was carried out in accordance with the permissions of the Instituto Chico Mendes de Conservação da Biodiversidade (ICMBio), number 55911‐3.

## 3 RESULTS

We recorded 101 fish species across 242 transects, representing 39 families (Table 1). Of these, 79 species (78.2%) were recorded during daytime and 39 species (38.6%) at night. A total of 62 species (61.4%) were exclusively observed during the day, 22 species (21.8%) only at night and 17 species (16.8%) were recorded in both periods. Percentages refer to the total species richness observed across all surveys. Five species accounted for more than 50% of all individuals recorded during daytime UVCs: *Coryphopterus glaucofraenum* (15.2%), *Stegastes fuscus* (13.3%), *H. aurolineatum* (12.2%), *Serranus baldwini* (6.8%) and *Holocentrus adscensionis* (5.6%). During night‐time UVCs, five species together represented over 90% of all individuals recorded: *H. aurolineatum* (70.8%), *H. adscensionis* (9.3%), *Pareques lineatus* (5.2%), *Phaeoptyx pigmentaria* (4.6%) and *Gymnothorax moringa* (1.2%). Percentages refer to the proportion of total individuals recorded in each period.

**TABLE 1:** Comparative fish species recorded during diurnal and nocturnal visual census.

Family	Species	Day		Night	
		Mean	SD	Mean	SD
Acanthuridae	*Acanthurus bahianus*	1.33	2.03		
	*Acanthurus chirurgus*	1.22	1.77		
	*Acanthurus coeruleus*	0.19	0.56		
Apogonidae	*Apogon americanus*			0.08	0.30
	*Apogon planifrons*			0.21	0.62
	*Apogon pseudomaculatus*			0.25	0.85
	*Apogon quadrisquamatus*			0.05	0.39
	*Astrapogon puncticulatus*			0.28	0.93
	*Phaeoptyx pigmentaria*			1.46	3.66
Blenniidae	*Parablennius marmoreus*	0.10	0.41		
	*Parablennius pilicornis*	0.13	0.43		
Bothidae	*Bothus ocellatus*	0.31	0.73		
Carangidae	*Caranx latus*	0.02	0.20		
	*Caranx ruber*	0.02	0.14		
Chaenopsidae	*Emblemariopsis signifer*	0.09	0.56		
Chaetodontidae	*Chaetodon sedentarius*	0.93	1.06		
	*Chaetodon striatus*	1.58	1.51		
Dactylopteridae	*Dactylopterus volitans*	0.53	1.17		
Diodontidae	*Chilomycterus spinosus spinosus*	0.38	0.70		
	*Diodon holocanthus*			0.01	0.09
	*Diodon hystrix*	0.04	0.19	0.07	0.25
Epinephelidae	*Epinephelus marginatus*	0.10	0.30	0.01	0.12
	*Hyporthodus niveatus*	0.01	0.10		
	*Mycteroperca acutirostris*	0.13	0.33	0.02	0.15
	*Mycteroperca interstitialis*	0.04	0.19	0.01	0.12
Fistulariidae	*Fistularia petimba*	0.02	0.14		
Gobiidae	*Coryphopterus glaucofraenum*	8.90	11.65		
	*Elacatinus figaro*	1.18	1.76		
	*Gnatholepis thompsoni*	0.28	0.79		
Gymnuridae	*Gymnura altavela*			0.01	0.09
Haemulidae	*Anisotremus virginicus*	0.39	1.07	0.14	0.46
	*Haemulon aurolineatum*	7.14	11.50	22.51	26.49
	*Haemulon parra*			0.02	0.19
	*Haemulon plumierii*	0.73	2.79	0.25	0.61
	*Haemulon steindachneri*			0.04	0.27
Holocentridae	*Holocentrus adscensionis*	3.27	5.38	2.94	2.37
	*Myripristis jacobus*			0.03	0.17
	*Plectrypops retrospinis*			0.01	0.09
	*Sargocentron bullisi*			0.31	0.87
Kyphosidae	*Kyphosus vaigiensis*	0.11	0.57		
Labridae	*Bodianus pulchellus*	0.42	0.71		
	*Bodianus rufus*	0.20	0.55		
	*Cryptotomus roseus*	0.42	1.15		
	*Halichoeres brasiliensis*	0.12	0.40		
	*Halichoeres poeyi*	2.04	2.02		
	*Scarus zelindae*	0.21	0.89		
	*Sparisoma amplum*	0.09	0.28		
	*Sparisoma axillare*	0.26	0.71		
	*Sparisoma frondosum*	0.52	1.34		
	*Sparisoma radians*	0.02	0.14		
	*Sparisoma tuiupiranga*	1.12	1.61		
Labrisomidae	*Labrisomus nuchipinnis*	0.03	0.17		
	*Malacoctenus delalandii*	0.14	0.38		
	*Malacoctenus triangulatus*	0.03	0.17		
Lutjanidae	*Ocyurus chrysurus*	0.02	0.14	0.02	0.15
Malacanthidae	*Malacanthus plumieri*	0.05	0.21		
Monacanthidae	*Aluterus scriptus*	0.01	0.10		
	*Cantherhines macrocerus*	0.05	0.21		
	*Cantherhines pullus*	0.57	0.73		
	*Stephanolepis hispida*	0.03	0.22		
Mullidae	*Pseudupeneus maculatus*	1.71	3.58		
Muraenidae	*Enchelycore nigricans*			0.01	0.09
	*Gymnothorax miliaris*	0.01	0.10	0.01	0.09
	*Gymnothorax moringa*	0.07	0.25	0.37	0.61
	*Gymnothorax vicinus*			0.03	0.21
Narcinidae	*Narcine brasiliensis*			0.01	0.12
Ogcocephalidae	*Ogcocephalus vespertilio*	0.05	0.21	0.08	0.35
Ophichthidae	*Myrichthys ocellatus*	0.02	0.14	0.01	0.09
Ostraciidae	*Acanthostracion polygonius*	0.14	0.43		
	*Acanthostracion quadricornis*	0.03	0.17		
Pempheridae	*Pempheris schomburgkii*			0.22	1.62
Pomacanthidae	*Centropyge aurantonotus*	0.13	0.42		
	*Holacanthus tricolor*	0.03	0.17		
	*Pomacanthus arcuatus*	0.01	0.10		
	*Pomacanthus paru*	0.27	0.54		
Pomacentridae	*Abudefduf saxatilis*	2.56	5.75		
	*Chromis flavicauda*	0.04	0.19		
	*Chromis jubauna*	0.01	0.10		
	*Azurina multilineata*	0.81	2.13		
	*Stegastes fuscus*	7.79	9.12		
	*Stegastes pictus*	1,95	3,60		
	*Stegastes variabilis*	0.01	0.10		
Priacanthidae	*Priacanthus arenatus*	0.57	2.34		
Rhinobatidae	*Zapteryx brevirostris*			0.15	0.58
Sciaenidae	*Eques lanceolatus*			0.02	0.15
	*Odontoscion dentex*	0.02	0.14	0.11	0.57
	*Pareques lineatus*	0.49	1.21	1.67	1.91
Scorpaenidae	*Scorpaena brasiliensis*			0.01	0.09
	*Scorpaena isthmensis*	0.05	0.26	0.30	0.88
	*Scorpaena plumieri*	0.03	0.17	0.01	0.12
	*Scorpaenodes tredecimspinosus*			0.01	0.17
Serranidae	*Rypticus bistrispinus*			0.03	0.17
	*Serranus baldwini*	3.95	5.76		
	*Serranus phoebe*	0.01	0.10		
Sparidae	*Calamus penna*	0.02	0.14		
	*Diplodus argenteus*	1.63	5.92		
Syngnathidae	*Hippocampus reidi*	0.01	0.10		
Synodontidae	*Synodus intermedius*	0.10	0.30		
	*Synodus synodus*	0.33	0.65		
Tetraodontidae	*Canthigaster figueiredoi*	0.18	0.46		
	*Sphoeroides camilla*	0.04	0.19		

*Note*: Values are average ± standard deviation per 40 m^2^.

Abbreviation: SD, standard deviation.

### 3.1 Reef fish assemblage

**FIGURE 1:**
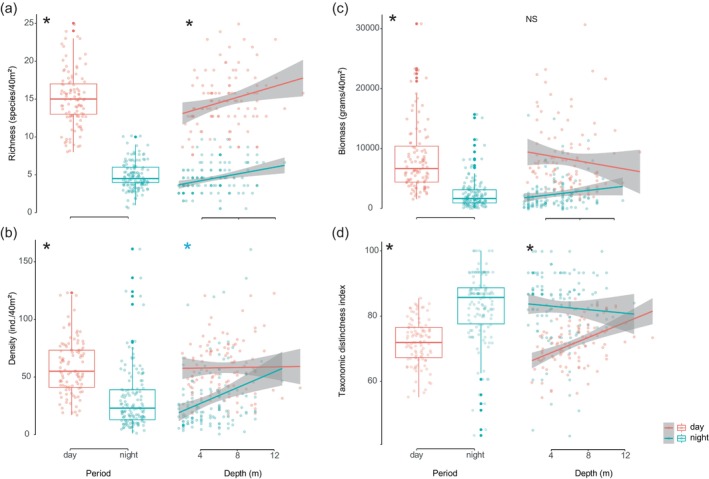
Comparative richness (a), density (b), biomass (c) and taxonomic distinctness index (d) of diurnal and nocturnal reef fish and the relationship between each metrics with depth (dots = samples, bar = median, box = first/third quartiles and whiskers = 1.5 * interquartile range, black asterisk indicates significant differences between periods, and blue asterisk indicates only to the night period).

Three community metrics describing reef fish assemblage structure – species richness (species/40 m^2^), density (individuals/40 m^2^) and biomass (grams/40 m^2^) – varied significantly between daytime and night‐time periods (Figure 1; Table 2). Daylight samples showed higher average values of fish richness, density and biomass per transect compared to nocturnal samples (Figure 1a–c). The diurnal samples showed lower taxonomic distinctness index compared to the nocturnal samples (Figure 1d). Diurnal and nocturnal species richness were positively correlated with the depth gradient; only nocturnal species density was positively correlated with depth, but no effect was detected with biomass (Figure 1; Table 2). The taxonomic distinctness index was positively related with the depth gradient during the day and negatively related during the night (Figure 1), indicating that diurnal species belonged to closely related taxonomical groups.

**TABLE 2:** Results of mixed‐effects models evaluating differences in reef fish assemblage metrics – species richness, total density, biomass, taxonomic distinctness (Δ^+^) and functional diversity indices (functional richness—FRic, functional evenness—FEve, functional divergence—FDiv and functional dispersion—FDis) – in relation to period of day and depth.

	Chisq	Richness			Density	
		df	*p* (>Chisq)	Chisq	df	*p* (>Chisq)
Period (day/night)	507.225	1	<0.001*	705.54	1	<0.001*
Depth	14.624	1	<0.001*	163.11	1	<0.001*
Period×depth	1.709	1	0.191	178.19	1	<0.001*

	Chisq	Biomasss			Taxonomic distinctness index	
		df	*p* (>Chisq)	Chisq	df	*p* (>Chisq)
Period (day/night)	115.006	1	<0.001*	90.901	1	<0.001*
Depth	0.765	1	0.382	3.934	1	0.050*
Period×depth	2.818	1	0.093	8.895	1	0.002*

	Chisq	FRic			FEve	
		df	*p* (>Chisq)	Chisq	df	*p* (>Chisq)
Period (day/night)	63.102	1	<0.001*	1.395	1	0.238
Depth	9.732	1	0.001*	0.067	1	0.795
Period×depth	0.050	1	0.824	2.114	1	0.146

	Chisq	FDiv			FDis	
		df	*p* (>Chisq)	Chisq	df	*p* (>Chisq)
Period (day/night)	60.322	1	<0.001*	23.721	1	<0.001*
Depth	1.272	1	0.259	0.001	1	0.979
Period×depth	5.799	1	0.016*	1.103	1	0.294

*Note:* Models included site as a random factor. Generalized linear mixed models (GLMM) were used for richness, density and biomass; linear mixed models (LMM) were applied to the remaining metrics. The significance values indicated by *.

**FIGURE 2:**
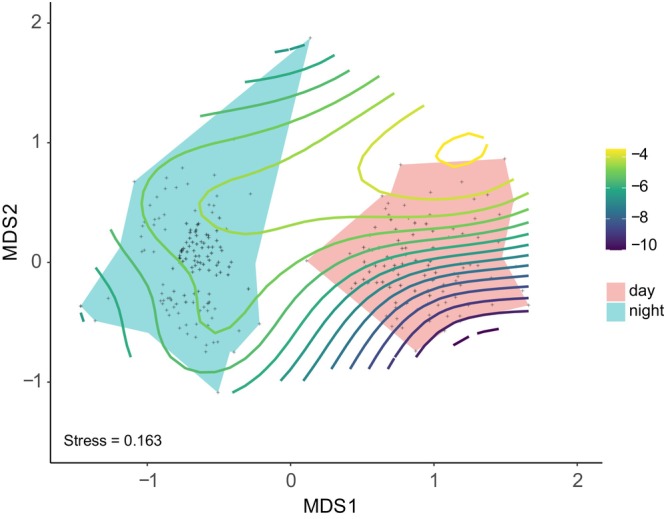
Non‐metric multidimensional scaling showing the influence of the depth variable on diurnal and nocturnal reef fish assemblage composition. (dots = samples, isolines = depth gradient, colours = diurnal or nocturnal assemblage).

Depth influenced the structure of the reef fish assemblage during daytime (PERMANOVA, df = 52; pseudo*‐F*_3_ = 1.37; *p* = 0.004), but had no effect on the structure of the assemblage at night (PERMANOVA, df = 35; pseudo*‐F* = 0.99; *p* = 0.51). Diurnal species composition showed differences on their abundances based on depth and had no overlap with the nocturnal species composition (Figure 2).

**FIGURE 3:**
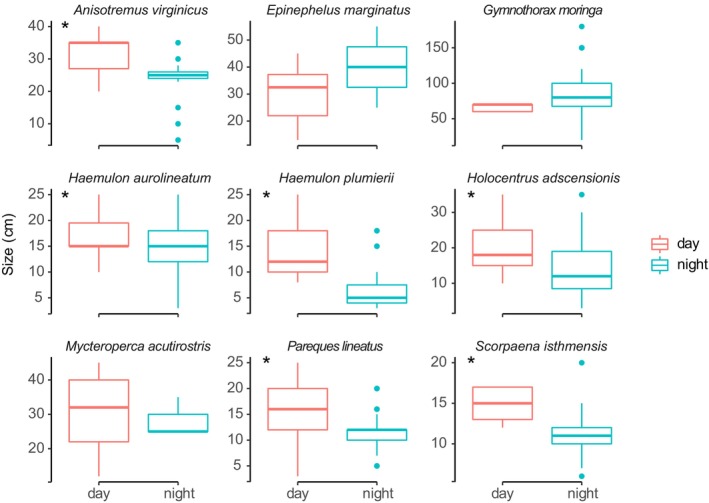
Comparative body size of reef fish species that occurred in both periods (day and night). (Bar = median, box = first/third quartiles and whiskers = 1.5 * interquartile range; black asterisk indicates significant differences).

The diurnal fish assemblage had a larger average individual size for many species compared to the nocturnal assemblage (ANOVA, *p* < 0.05; Figure 3; Table 3).

**TABLE 3:** Results of analysis of variance (ANOVA) for species size among periods (day and night).

Species	*t* value	*p* (>|*t*|)
*Anisotremus virginicus*	−3.383	0.002
*Epinephelus marginatus*	0.881	0.399
*Gymnothorax moringa*	0.887	0.379
*Haemulon aurolineatum*	−2.990	0.003
*Haemulon plumierii*	−7.1034	<0.001
*Holocentrus adscensionis*	−10.569	<0.001
*Mycteroperca acutirostris*	0.097	0.924
*Pareques lineatus*	−5.350	<0.001
*Scorpaena isthmensis*	−2.526	0.017

### 3.1 Functional role of reef fish assemblage

**FIGURE 4:**
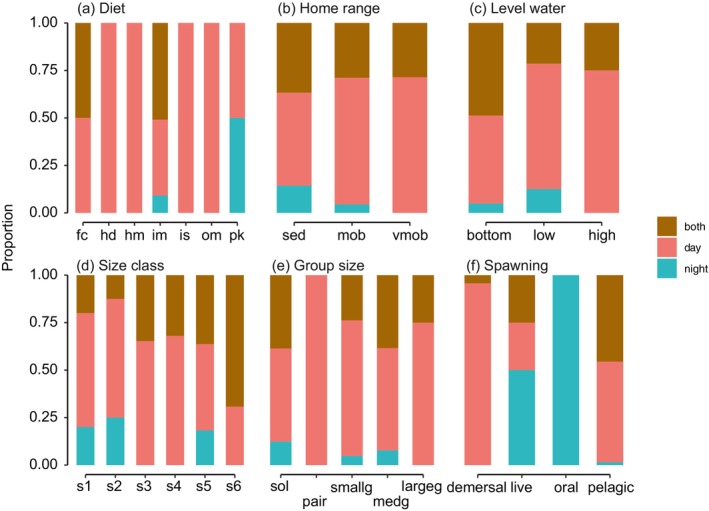
Comparative proportion of traits among diurnal species (pink), nocturnal species (blue) and species active in both periods (brown). (a) Diet: Fc, piscivores; hd, herbivore‐detritivores; im, mobile benthic invertebrates; is, sessile invertebrate feeders; om, omnivores; pk, planktivores. (b) Home range: sed, sedentary; mob, mobile; vmob, very mobile. (c) Level water: bottom, low and high. (d) Size class: s1: 0–7 cm; s2: 7.1–15 cm; s3: 15.1–30 cm; s4: 30.1–50 cm; s5: 50.1–80 cm; s6: >80 cm. (e) Group size: sol, solitary; pair, pairing; smallg, small groups; medgroup, medium groups; largeg, large groups. (f) Spawning: demersal, live, oral and pelagic.

**FIGURE 5:**
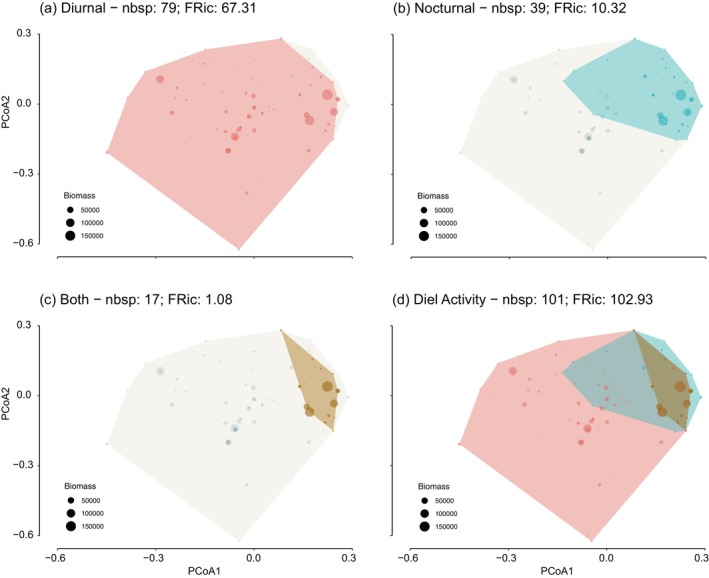
Functional space filled by diurnal fish species (a), nocturnal fish species (b), species that were recorded in both periods (c) and diel activity (24‐h cycle – all species observed) (d). (Convex hull and circles colours: grey = all samples, pink = diurnal, blue = nocturnal and brown = both diurnal and nocturnal; circle is a sample, and its size is proportional to its total standing biomass).

The diurnal assemblage includes all traits, except oral spawning (Figure 4). Strictly nocturnal species were mainly composed of mobile benthic invertebrate feeders and planktivores, with a strong representation of apogonids, most of which are oral incubators. The species recorded as being active in both periods were primarily piscivores and mobile benthic invertebrate feeders (Figure 4). The diurnal reef fish assemblage, which was comparatively richer as expected, occupied the largest portion of the total multidimensional functional volume (65.4%). The nocturnal assemblage had 10% of total volume, whereas species that are active in both periods have only 1.1%, but each of them presented particularities on traits categories (Figure 5).

**FIGURE 6:**
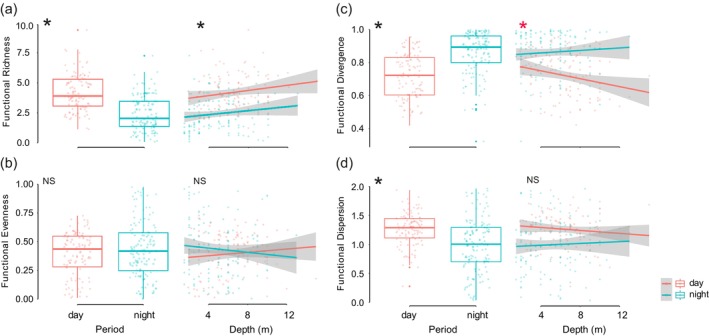
Diurnal and nocturnal functional richness (a), functional evenness (b), functional divergence (c) and functional dispersion (d), along with their relationship with depth. (Circles = samples, bar = median, box = first/third quartiles and whiskers = 1.5 × interquartile range; black asterisk represents statistical differences in both periods, and red asterisk represents only to the day period).

Diurnal functional richness (FRic) was higher than nocturnal FRic (Table 2), and both of them were positively influenced by depth (Figure 6a). Functional evenness (FEve) values were similar between both assemblages and were not influenced by depth (Figure 6b; Table 2). Diurnal functional divergence (FDiv) values were lower than nocturnal FDiv values and were negatively influenced by depth (Figure 6c). Diurnal functional dispersion (FDis) values were highest compared to nocturnal FDis values, and neither was influenced by the depth gradient (Figure 6d; Table 2).

## 4 DISCUSSION

Although significant advances have been made in understanding reef fish dynamics and functional ecology during the daytime across various spatial and temporal scales (Ferreira et al., 2001; Floeter et al., 2005; Leprieur et al., 2016), nocturnal reef fish communities remain poorly understood. For the subtropical reef fish assemblages studied, the diurnal assemblage, as expected, exhibited higher species richness, density and biomass. Few studies have explicitly compared assemblage metrics between diurnal and nocturnal reef fish assemblages using standardized sampling (Azzurro et al., 2007; Brewin et al., 2016; Harvey, Butler, et al., 2012; Harvey, Dorman, et al., 2012; Hinojosa et al., 2020; Jones et al., 2024; Myers et al., 2016). Our finding supports previous studies showing that diurnal communities tend to harbour more species, greater numbers of individuals and higher biomass compared to nocturnal assemblages (Azzurro et al., 2007; Cardoso et al., 2020; Harvey, Butler, et al., 2012; Nagelkerken et al., 2000). The depth gradient significantly influenced some of these metrics. Individuals occurring in both diurnal and nocturnal assemblages differed in body size, with comparatively larger individuals observed during the diurnal period. Species traits also varied between the two periods, with diurnal species occupying a larger proportion of the multidimensional trait space.

These differences were consistent across our three study sites, suggesting an expected similarity among them. Most tropical and temperate reef fish are diurnal (Helfman, 1986; Hobson, 1965), whereas only about 30% exhibit primarily nocturnal activity (Helfman, 1978). The diurnal period in reefs, as in many natural systems at earth surface, is the most productive in terms of energy and material fluxes (Morais et al., 2025). Critical ecological processes are sustained by the interaction between different components of biodiversity and the abiotic environment, with fish communities exhibiting higher metrics during the daytime (e.g., species richness, density and biomass). The higher number of fish species observed during the day also suggests a more differentiated use of resources, reflecting functional complementarity and biological facilitation among species (Brandl et al., 2019; Hooper et al., 2012).

At the subtropical reefs studied, 21.8% of species were exclusively found active at night, an assemblage mainly composed by Apogonidae (cardinalfishes), Holocentridae (soldierfishes and squirrelfishes), Haemulidae (grunts) and Pempheridae (sweepers), the most widespread and conspicuous nocturnal reef fish families (Kulbicki et al., 2013; Schmitz & Wainwright, 2011). Diurnal reef fish assemblages are structured by different factors depending on the spatial scale analysed: at the local scale by depth (Ferreira et al., 2001), structural complexity (Brokovich et al., 2006; Dominici‐ Arosemena & Wolff, 2006) and wave exposure (Lecchini et al., 2003); and at regional and evolutionary scales, by sea surface temperature (SST) and primary productivity (Floeter et al., 2005) (Leprieur et al., 2016). Nocturnal assemblages are clearly subjected to the same factors and environmental gradients; in this study, the depth gradient significantly influenced some fish metrics. The depth gradient is known to be also negatively related to primary productivity and positively related to predator abundance and resource competition (Friedlander & Parrish, 1998), which may influence reef fish species distribution during the day. Diurnal and nocturnal species richness were positively correlated with the depth gradient; however, only nocturnal species density showed a positive correlation with depth, and no significant effect of depth was detected on biomass. All shallow reefs along the Brazilian subtropical coast tend to support similar patterns in diurnal fish assemblages (Ferreira et al., 2001; Floeter et al. 2008). However, these patterns are likely to shift with increasing depth, primarily due to colder water temperatures in the deeper habitats (Gragnolati et al., 2024), limiting many fish species that occur in the subtropical realm of the Brazilian Southeastern coast, those that are widespread tropical affinity species (Pinheiro et al., 2018).

The higher taxonomic distinctness observed in nocturnal reef fish assemblages suggests that, at night, although species composition may be morphologically similar, the species are phylogenetically more distant from one another compared to those in the diurnal community.This may have been driven by the presence of elasmobranchs (such as *Narcine brasiliensis* and *Zapteryx brevirostris*), only sighted at night‐time, whereas diurnal assemblages were largely dominated by Perciformes (Luiz et al., 2015; Morais et al., 2017). These elasmobranchs are recognized by being more active at night, but their absence during daytime sampling is likely a result of intense fishing (Bender et al., 2014). Indeed, the population of several species of sharks and rays has been dramatically reduced in the region in the past decades (Fogliarini et al., 2021). Taxonomic distinctness is a metric independent of sample size or sampling effort (Clarke & Warwick, 1998), whereas taxonomic structure of the fish assemblage is considered to be as important as species richness to understand the biodiversity of natural assemblages (Clarke & Warwick, 2001). Therefore, closely related species in an assemblage must be regarded as less ‘taxonomically diverse’ than a similar rich assemblage of more distantly related species, for example, all belonging to different orders (Clarke & Warwick, 2001).

Diurnally active species were representative of all diet categories, showing greater trophic variability. Apart from herbivorous fishes, which constitute a major portion of reef biomass during the day (Cordeiro et al., 2015) but are absent at night – as are many omnivores – the nocturnal fish assemblage (species richness: 76.9%; total abundance: 85.3%) consisted primarily of benthic invertivores. An iconic group of nocturnal fishes, the apogonids and soldierfishes, exhibited clear habitat partitioning. *Apogon* spp. are mobile invertebrate feeders that can opportunistically forage on planktonic invertebrates. They were generally distributed from the mid to deep zones of the studied rocky reefs. The genus *Astrapogon* specifically inhabits the interface between the reef and sandy bottoms. The planktivore *P. pigmentaria*, unlike the other apogonids, forages in the water column 2–3 m above the bottom, primarily in the deeper parts of the reef or at the reef–sand interface. Many of the smaller *Scorpaena* spp. are also sand‐dwelling at night, foraging on benthic and planktonic invertebrates that emerge in abundance after dark. The soldierfish *H. adscensionis* is abundant even during the day but becomes more active at night. Together with *Myripristis jacobus* and *Sargocentron bullisi*, these species were found foraging from shallow areas to the reef–sand interface. Haemulids, especially *H. aurolineatum*, one of the most abundant species during the day, forage solitarily on sand flats at night. During the day, however, they form schools and may feed on plankton wherever and whenever available. Other nocturnal species, such as *Pempheris* schomburgkii, *Pareques* lineatus and *Odontoscion* dentex, are diurnal cave dwellers that emerge at night to forage on plankton and other benthic invertebrates. Pufferfishes, such as *Diodon* spp., although occasionally active during the day, are more commonly nocturnal, foraging on urchins and molluscs. Finally, groupers and sea basses were apparently active during our night surveys but are generally considered diurnal or crepuscular predators (Harmelin‐Vivien & Harmelin, 2022). Because our surveys were conducted during the early hours of the night, it is possible that these species were still actively foraging. Morays are also typically crepuscular and nocturnal predators.

Body size is one of the most fundamental attributes of fishes and can directly influence their abundance (White et al., 2007), home range (Welsh et al., 2013), dispersal (Luiz et al., 2012) and functional capabilities (Welsh & Bellwood, 2014). Our results demonstrate that species registered in both diurnal and nocturnal periods were larger during daytime than at night. This could indicate that these species are partitioning their niches according to ontogeny (Pereira et al., 2014), whereby larger (older) individuals use resources during daytime, and smaller (younger) individuals explore resources at night. During the day, as mentioned, some of these species (especially Haemulidae and Holocentridae) were observed more frequently forming schools or resting in groups possibly to avoid predation. The movement of fish from day to night among different habitats (e.g., sand bottom or water column) may be influenced by the presence of predators (Harvey, Butler, et al., 2012), which would be a response to avoid risky areas (Hammerschlag et al., 2010). In the subtropical rocky reefs studied, the landscape of habitats is very simple, with rocky shores with an average slope of 40 degrees ending on sand bottom. Fish movement is largely restricted to reef zones (shallow to deep) and the reef–sand interface.

The ability of each species to actively participate in ecological processes depends on specific biological traits linked to food acquisition and locomotion (Villeger et al., 2010; Winemiller, 1991) and is closely related to the period of the day at which one is active (Pereira et al., 2014). The functional richness of both assemblages was positively related with depth, as opposed to what was found in central‐western Pacific islands (Yeager et al., 2017). It would be expected that functional richness is negatively related to deeper reefs, as shallow areas present higher primary and benthic productivity (Friedlander & Parrish, 1998; Klumpp & McKinnon, 1989). A possible explanation is that our study site exhibits a relatively narrow depth gradient (up to 12 m) compared to reefs of the central‐western Pacific Ocean islands (up to 29 m). Additionally, in our study area, deeper zones tend to have more heterogeneous topography than shallow zones (Ferreira et al., 2001), which may support a greater diversity of species with different functional attributes.

The regularity in the distribution of species biomass in the functional space (functional evenness) was similar between diurnal and nocturnal assemblages and was not influenced by depth. This suggests that both assemblages have high heterogeneity of species biomass distribution in the functional space, probably because some parts of the trait space are empty whereas others are densely occupied (Mouchet et al., 2010). Similarly, functional dispersion during daylight was higher than at night, which means that diurnal species present more extreme attributes in relation to the most abundant species on the assemblages (Laliberte & Legendre, 2010).

Biomass of diurnal and nocturnal reef fish assemblage was dominated by different species, characterized by different traits, reflecting the high functional divergence observed. Although some functional entities are shared between diurnal and nocturnal assemblages, several functional entities are exclusive to one or other assemblage. Functional divergence was higher in the nocturnal assemblage compared to the diurnal one, suggesting a greater degree of niche differentiation among nocturnal species and potentially reduced competition (Mouchet et al., 2010). This result is in accordance with the higher taxonomic distinctiveness found for the nocturnal assemblage once taxonomic distinct species are more likely to present different traits than closely related species. Although FDiv was lower in the diurnal assemblage, it was higher in shallow areas than in deeper ones, indicating that diurnal reef fishes were more variable in shallow areas. This is also a result of the higher contribution of herbivorous fish in these habitats (Cordeiro et al., 2015; Ferreira et al., 2001). A prominent differentiation between diurnal and nocturnal assemblages was the presence of herbivorous and omnivorous fishes during daylight, and mainly mobile invertivorous and zooplanktivorous fishes at night, corroborating with previous studies (Harvey, Butler, et al., 2012; Hobson, 1965; Jones et al., 2024; Newman & Williams, 2001). Nocturnal ecological functions may be underrepresented or underestimated because the ecological attributes of nocturnal species have been derived from diurnal observations, which could introduce bias compared to observations during their active period.

Ecosystem functioning has been a key focus in contemporary management initiatives (Bellwood et al., 2019). Although there is broad consensus that biodiversity can promote ecosystem functioning (Duffy et al., 2017), and that the loss of key species threatens reef functioning in the Anthropocene (Hughes et al., 2017), there remains a significant gap in knowledge regarding the nocturnal reef fish community and the potential role of species identity in coral reef functioning. In this study, we present, for the first time in South Atlantic coastal reefs, measures of taxonomic, functional and phylogenetic diversity for fish assemblages. We aimed to unveil *who* constitutes the nocturnal assemblage in subtropical reefs, and how it differs from the diurnal one. Among a hundred reef fish species sampled during day and night, nocturnal assemblage makes up 20% of the total, filling up 10% of multifunctional space. Most diurnal fishes are small in size and have low mobility, being carnivorous in general. Although most nocturnal fish have little economic value, many small ones like apogonids, are part of the cryptobenthic community, integrating a key role in coral reef trophodynamics by cycling trophic energy provided by microscopic prey to larger consumers (Brandl et al., 2018). Cryptobenthic fishes transform energy and nutrients into biomass that is readily accessible and rapidly consumed by a wide range of larger predators (Brandl et al., 2018; Depczynski & Bellwood, 2006; Goatley et al., 2017). Also, the high abundance of cryptobenthic larvae has critical function to reef trophodynamics via rapid growth and extreme mortality, producing almost 60% of consumed reef fish biomass (Brandl et al., 2018). Large fishes, such as groupers and basses that can also be active at night, probably can contribute to nutrient cycling between night and day (Schiettekatte et al., 2022), meaning that foraging at night in different habitats, others than those during day, those species can contribute to nutrient cycling between habitats and different reef substrates. The same can be said for haemulids or puffers, which exhibit significant mobility within the reef either during the day or night and can significantly contribute to energy connectivity among habitats and periods (Meyer & Schultz, 1985; Nagelkerken et al., 2000). The ontogenetic separation in the activity periods of species active in both day and night, and their contribution to nutrient cycling, is still to be understood (Schiettekatte et al., 2022). Fish play important roles in ecosystem processes, primarily through the regulation of food webs and nutrient cycling, as well as in ecosystem services, such as providing biomass for human nutrition and cultural benefits (Holmlund & Hammer, 1999; Villéger et al., 2017). Ecosystem functions have intrinsic relationships with species identity and composition, interacting with the abiotic environment and mediating the rates of ecological processes (Brandl et al., 2019). Although we are beginning to understand the functional roles of diurnal reef fish assemblages (Bellwood et al., 2019), much work remains to be done to understand the role of nocturnal fish in reef processes.

## AUTHOR CONTRIBUTIONS

Conceptualization (M.B.L., C.E.L.F.), methodology and investigation (M.B.L., T.C.M., C.A.M.M.C.), formal analysis (M.B.L., T.C.M., C.A.M.M.C.); writing – original draft (M.B.L.,), writing – review and editing (M.B.L., T.C.M., C.A.M.M.C., C.E.L.F.).

## FUNDING INFORMATION

This research was funded by Costão Rochoso Project, a partnership with Petrobras (Programa Petrobras Socioambiental). C.E.L.F is supported by CNPq ‐ Conselho Nacional de Desenvolvimento Cientifico e Tecnologico (310291/2023‐0), and FAPERJ ‐ Fundação Carlos Chagas Filho de Amparo à Pesquisa do Estado do Rio de Janeiro (E‐26/201.026/2022). C.A.M.M.C. is supported by FAPERJ (E‐26/200.215/2023).

## Supporting information


**Figure S1.** Map of the study area showing the location of the three sampling sites within the coastal reef system: (a) Ilha dos Porcos, (b) Pedra Vermelha and (c) Anequim.

**Table S1.** Standard deviations of the random effect (site) and residuals from mixed‐effects models applied to reef fish assemblage metrics and functional diversity indices. Singular fits indicate that no variability was detected among sites.
