# Supplementary figures and images for: Period of the day drives distinctions in the taxonomic and functional structures of reef fish assemblages
Source: J Fish Biol. 2025 Sep 13;108(1):103–17. doi: 10.1111/jfb.70228 (PMC13033963; doi:10.1111/jfb.70228)

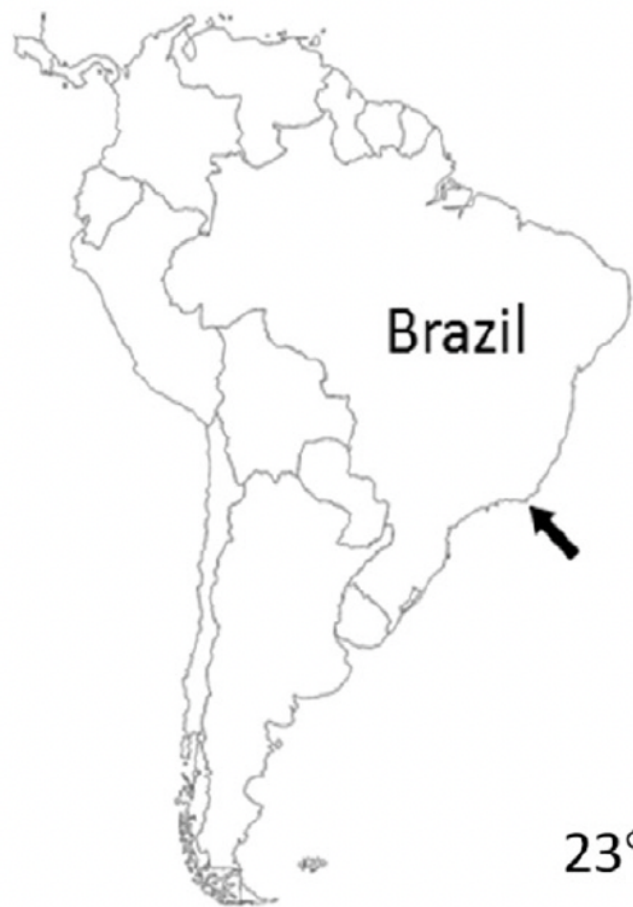

23° S

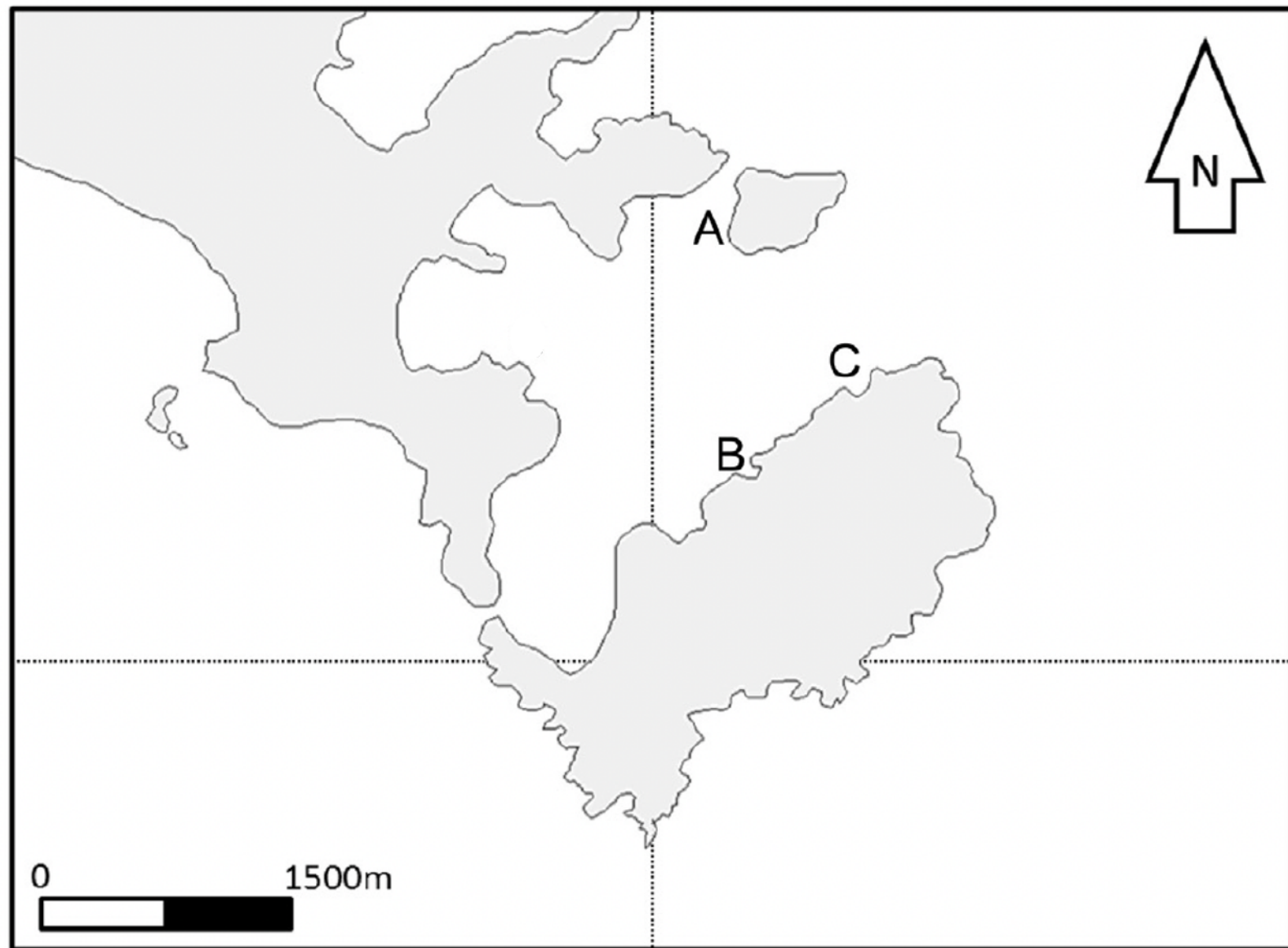

42° W

Supplement: Supplementary file 1 — Figure S1. Map of the study area showing the location of the three sampling sites within the coastal reef system: (a) Ilha dos Porcos, (b) Pedra Vermelha and (c) Anequim. [file JFB-108-103-s002.pdf]
