# Supplementary material for: Period of the day drives distinctions in the taxonomic and functional structures of reef fish assemblages
Source: J Fish Biol. 2025 Sep 13;108(1):103–17. doi: 10.1111/jfb.70228 (PMC13033963; doi:10.1111/jfb.70228)
Supplement: Supplementary file 2 — Table S1. Standard deviations of the random effect (site) and residuals from mixed‐effects models applied to reef fish assemblage metrics and functional diversity indices. Singular fits indicate that no variability was detected among sites. [file JFB-108-103-s001.docx]

Supplementary Table S1: Standard deviations of the random effect (site) and residuals from mixed-effects models applied to reef fish assemblage metrics and functional diversity indices. Singular fits indicate that no variability was detected among sites.

| **Metric** | **Model type** | **Site Std.Dev.** | **Residual Std.Dev.** | **Notes** |
| --- | --- | --- | --- | --- |
| Species richness | GLMM (Poisson) | 0,04599 |  | Std.Dev. on log scale |
| Fish density | GLMM (Poisson) | 0,1264 |  | Std.Dev. on log scale |
| Biomass | GLMM (Tweedie) | 0,1738 |  | Fitted with glmmTMB |
| Taxonomic distinctness (Δ⁺) | LMM | 1,469 | 8,958 | Moderate spatial variability |
| Functional richness (FRic) | LMM | 0,5551 | 1,455 | Moderate spatial variability |
| Functional evenness (FEve) | LMM (singular) | 0 | 0,2207 | No site-level variance (singular fit) |
| Functional divergence (FDiv) | LMM (singular) | 0 | 0,1363 | No site-level variance (singular fit) |
| Functional dispersion (FDis) | LMM | 0,04293 | 0,3995 | Low but non-zero site-level variance |
